# Relationship Between the Lipid Accumulation Product Index and Arterial Stiffness in the Chinese Population With Hypertension: A Report From the China H-Type Hypertension Registry Study

**DOI:** 10.3389/fcvm.2021.760361

**Published:** 2022-01-21

**Authors:** Yumeng Shi, Lihua Hu, Minghui Li, Wei Zhou, Tao Wang, Lingjuan Zhu, Huihui Bao, Ping Li, Xiaoshu Cheng

**Affiliations:** ^1^Department of Cardiovascular Medicine, The Second Affiliated Hospital of Nanchang University, Nanchang, China; ^2^Jiangxi Provincial Cardiovascular Disease Clinical Medical Research Center, Nanchang, China; ^3^Department of Cardiovascular Medicine, Peking University First Hospital, Beijing, China; ^4^Department of Cardiovascular Medicine, Inner Mongolia People's Hospital, Hohhot, China; ^5^Center for Prevention and Treatment of Cardiovascular Diseases, The Second Affiliated Hospital of Nanchang University, Nanchang, China

**Keywords:** lipid accumulation product index, brachial-ankle pulse wave velocity, arterial stiffness, hypertension, insulin resistance

## Abstract

**Background:**

Lipid accumulation product (LAP) index, as a new index to predict cardiovascular disease (CVD), has attracted the attention of many researchers. The relation of the LAP index with brachial-ankle pulse wave velocity (baPWV) has been evaluated in several previous studies and yielded inconsistent results. This study aimed to investigate the association between the LAP index and arterial stiffness in the Chinese population with hypertension.

**Methods:**

We conducted a cross-sectional analysis in 4,926 Chinese population with hypertension (aged 64.42 ± 9.44 years). The LAP index was developed from population-based frequency plots of adult waist circumferences and circulating triglyceride concentrations according to men and women. Arterial stiffness was determined by measuring baPWV.

**Results:**

The multivariate linear regression analyses showed that the LAP index was independently and positively associated with baPWV [beta coefficients *(*β*)*, 24.10 cm/s; 95% CI: 5.72, 42.49 cm/s]. Consistently, the multivariate logistic regression analyses showed a positive association between the LAP index risk of elevated baPWV (>75th percentile) [odds ratio (OR), 1.19; 95% CI 1.01, 1.41]. A restricted cubic spline showed that a significant linear association existed between the LAP index and baPWV. In different subgroups of diastolic blood pressure (DBP), there is interaction between the LAP index and baPWV (DBP <90, 90–99, ≥100 mm Hg; *p*-interaction = 0.006).

**Conclusion:**

LAP is significantly positively associated with baPWV and elevated baPWV in Chinese hypertensive adults and especially among participants with DBP ≤ 99 mm Hg.

## Background

Brachial-ankle pulse wave velocity (baPWV) is the most common method for measuring arterial stiffness in the Asian population (1) and it is widely used in many epidemiological studies because of its non-invasive, convenient, and inexpensive (2–4). It is reported that metabolic syndrome (5), cardiovascular disease (CVD) (6), stroke (7), mortality (8), and kidney disease (9) are all related to elevated baPWV. In China, hypertension is a common health problem and a major pathogenic factor of arterial stiffness, which can cause a high proportion of hypertension and cardiovascular disability (10, 11). According to the latest national hypertension survey of 451,755 adults in China from 2012 to 2015, the prevalence of hypertension is 27.9% (12). Estimates suggest that the total number of hypertension patients among Chinese adults is 244.5 million (13). Therefore, a better understanding of the potential risk factors of increased arterial stiffness among hypertensive patients may help to prevent the onset of arterial stiffness and related CVDs.

The lipid accumulation product (LAP) index, as a new index to predict CVD, has attracted the attention of many researchers. LAP was proposed as a better continuous marker/index to describe lipid overaccumulation in relationship to central obesity and metabolic risks (14) and was shown to outperform body mass index (BMI) in the identification of CVD risk (15) and diabetes (16). The calculation method of the LAP index varies with gender: males: [(waist circumference (WC)-65) × triglycerides (TGs)]; women: [(WC-58) × TGs] (17). There are only a few previous studies on LAP and arterial stiffness, but their conclusions are inconsistent (18–20). Moreover, all the above studies were carried out in healthy adults and few people in hypertensive patients pay attention to the effect of LAP on arterial stiffness.

In order to fill the gap of the above study, this cross-sectional analysis uses the data of the Chinese H-type Hypertension Registration Study to evaluate the association between the LAP index and arterial stiffness in the Chinese population with hypertension and explore whether there are effect modifiers that can change the relationship between them.

## Methods

### Subject Population and Design

This study was based on the China H-type Hypertension Registry Study (Registration number: ChiCTR1800017274); briefly, the China H-type Hypertension Registry Study is an ongoing, real-world, observational study conducted in Wuyuan, Jiangxi province of China. The full details with respect to the design and rationale of this study were extensively described elsewhere (21). Participants recruited in 2018 were consecutively enrolled if they: (1) were aged ≥18 years and (2) had hypertension, defined as seated resting blood pressure ≥140/90 mm Hg or self-reported use of antihypertensive medications. The participants were excluded when subjects with neurological abnormalities were unable to follow-up according to this study protocol under the Declaration of Helsinki conducted this study. Approval of this study protocol was provided by the Ethics Committees of the Biomedical Institute of Anhui Medical University (Approved No. of Ethic Committee: CH1059). Before each participant entered this study, they all signed the informed consent.

A total of 5,233 subjects had baPWV measurements taken at baseline. After excluding those with ankle-brachial indexes (ABI) <0.90 (*n* = 133) (22) or using lipid-lowering medications (*n* = 174), finally, 4,926 patients were selected for this study (Supplementary Figure 1).

### Data Collection

Standardized questionnaires were used to obtain demographic factors, lifestyle behaviors (smoking and alcohol consumption), disease history, family history of diseases, and medication use at baseline. Current smoking was defined as smoking ≥1 cigarette per day for 1 year or more or a cumulative smoking amount ≥360 cigarettes per year. Alcohol consumption was defined as drinking an average of at least two or more times a week over a year. At the same time, we also collected the anthropometric indices included weight, height, WC, and hip circumference (HC) of all the participants. The BMI was calculated as the body weight in kilograms/square of the height in meters (kg/m^2^). Blood pressure (BP) was measured in the sitting position using an electronic sphygmomanometer (Omron; Dalian, China). After a 10-min rest period, BP was measured three times and the average of the three measurements was used for final analyses.

### Brachial-ankle Pulse Wave Velocity Measurements

The baPWV (cm/s) and ABI were automatically measured simultaneously in the supine position after resting for more than 10 min, using an automatic waveform analyzer (BP-203RPE III device; Omron Health Care, Kyoto, Japan).

The specific measurement method is as follows: blood pressure cuff is wrapped on arms and ankles, the lower edge of the armband is located 2–3 cm above the cubital fossa transverse stripes, while the lower edge of the ankle band is located 1–2 cm above the medial malleolus. Furthermore, the baPWV value was calculated as the ratio of transmission distance from the brachium to ankle divided by the transit time:


$$\begin{array}{l} \text{baPWV}=\frac{\text{La }-\text{Lb }}{\Delta \text{ Tba}} \end{array}$$

*Lb* and *La* refer to the path length from suprasternal incision to the brachial muscle (*Lb*) and from suprasternal incision to the ankle (*La*), respectively. Δ*Tba* is the ankle-brachial artery pressure wave foot time difference. baPWV was measured twice automatically and the higher value of the left and right sides was used in the final analysis.

### Laboratory Tests

After an 8–10 h fasting period, all the blood samples were taken from the cubital vein and delivered to the Biaojia Biotechnology Laboratory, Shenzhen, China. Automatic clinical analyzers (Beckman-Coulter Canada, Inc., Mississauga, Canada) were used to measure biochemicals included creatinine, uric acid, plasma homocysteine (Hcy), fasting total cholesterol (TC), TGs, high-density lipoprotein cholesterol (HDL-C), and fasting plasma glucose (FPG). LAP was determined from WC (cm) plus TG (mmol/l) for men [(WC-65) × TG] and women [(WC-58) × TG] (23); to prevent non-positive values for LAP, any male WC values of 65 cm or less were revised upward to 66.0 cm and any female WC values of 58 cm or less were revised upward to 59 cm (15). The estimated glomerular filtration rate (eGFR) was calculated by the Chronic Kidney Disease (CKD) Epidemiology Collaboration formula (24).

### Statistical Analysis

Based on quartiles of LAP levels, the study population was divided into four groups. The characteristics were presented as mean (SD) and categorical variables were expressed as count (percentage). We compared baseline characteristics on quartiles of participants of LAP levels by the one-way ANOVA for continuous variables and the chi-squared test or the Fisher's exact test for categorical variables, as appropriate. The distribution of LAP was strongly skewed toward the left. Thus, we performed the ln-transformed (lnLAP) on the LAP index before the analysis.

The predictive value of baPWV in cardiovascular events is unavailable. In this study, elevated baPWV is defined as a value >75% of baPWV value and >2,059 cm/s. The Pearson's correlation coefficient was used to assess the association of the LAP index with cardiovascular risk factors. Beta coefficients (β) and 95% CI used to investigate the association between LAP and baPWV were calculated using the multivariate linear regression analysis; odds ratios (ORs) and 95% CI used to investigate the association between LAP and elevated baPWV in hypertensive participants were calculated using the multivariate logistic regression analysis for five models. These five models are as follows: model 1 adjusted only for age and sex; model 2 adjusted for prior covariates and BMI, systolic blood pressure (SBP), diastolic blood pressure (DBP), pulse rate, pulse pressure, smoking status, and drinking status; model 3 adjusted for these prior covariates plus fasting blood glucose (FBG), TC, HDL, low-density lipoprotein cholesterol (LDL-C), Hcy, uric acid, eGFR, diabetes mellitus, antihypertensive drugs, and antiplatelet drugs; model 4 adjusted for these prior covariates plus the types of antihypertensive drugs and duration of hypertension; and model 5 adjusted for these prior covariates plus glucose-lowering drugs. Variables known as traditional risk factors for stroke and the potential confounders were selected, if the effect estimates individually changed by at least 10% (25). To characterize the shape of the dose-response relationship of the LAP index with baPWV and elevated baPWV, we performed a generalized additive model (GAM) and a fitted smoothing curve (penalized spline method). As additional exploratory analyses, possible modifications of the association between the LAP index and baPWV were also evaluated for variables including sex (males vs. females), age (<65 vs. ≥65 years), BMI (<25 vs. ≥25 kg/m^2^), current smoking (no vs. yes), current drinking (no vs. yes), SBP (<140, 140–159, ≥160 mm Hg), DBP (<90, 90–99, ≥100 mm Hg), diabetes mellitus (no vs. yes), and eGFR (<60 vs. ≥60 ml/min/1.73 m^2^). In order to ensure the robustness of data analysis, we conducted the following sensitivity analysis: (1) We have exclude 59 cancer patients and discussed the relationship between LAP and baPWV in non-cancer participants and (2) We evaluated the relationship between LAP and baPWV in men and women, respectively.

Data were analyzed using the Empower (R; www.empowerstats.com; X&Y Solutions Incorporation, Boston, Massachusetts, USA) and the statistical package (R) (http://www.R-project.org, The R Foundation). Effects that met the 5% significance level were considered as statistically significant, with all the tests being two-sided.

## Results

### Baseline Characteristics

A representative sample of 4,926 participants of the China Hypertension Registry Study was eligible and enrolled in the analysis. From the study population, the mean age of those participants was 64.42 ± 9.44 years old and 2,440 (49.53%) people were men. The overall mean baseline LAP index was 40.65, ranging from 0.14 to 514.60 and mean baPWV was 1855.27 (414.58) cm/s. The baseline clinical characteristics of participants according to the quartile of the LAP index are given in Table 1. Compared with participants in the lowest quartile group, participants with the higher LAP index tended to be younger women, have diabetes mellitus and higher BMI, have a low prevalence of current smokers and drinkers, and have lower values of HDL and Hcy. Likewise, significant differences in biological parameters were observed among the groups. DBP, pulse rate, pulse pressure, FBG, TC, LDL, eGFR, and uric acid of the participants in the highest LAP index quartile were significantly higher than those in the first quartile. Moreover, the high LAP index groups had higher use of antihypertensive drugs, antiplatelet drugs, and glucose-lowering drugs (*p* < 0.05). In different genders, we describe the baseline characteristics according to the quartile of the LAP index (Supplementary Tables 3, 4). Supplementary Table 3 shows that there is no significant difference in Hcy and SBP among men in LAP four groups, but there are significant differences in other variables. Supplementary Table 4 shows that there is no significant difference in current drinking, SBP, Hcy, eGFR, and antiplatelet drugs among women in LAP four groups, but there are significant differences in other variables.

**Table 1:** Clinical characteristics of the study population according to LAP.

**Variable**			**InLAP**		***P-*value**
	**Quartile 1**	**Quartile 2**	**Quartile 3**	**Quartile 4**	
LAP range	0.14 to <14.75	14.75 to <29.79	29.79 to <52.83	52.83 to ≤ 514.60	<0.001
Participants	1,230	1,233	1,231	1,232	
Males, *N*	865 (70.33%)	620 (50.28%)	501 (40.70%)	454 (36.85%)	<0.001
DBP, mmHg	86.43 ± 11.22	88.86 ± 10.99	89.17 ± 10.59	91.15 ± 10.43	<0.001
Age, year	67.47 ± 8.81	65.25 ± 9.53	63.85 ± 9.20	61.10 ± 9.07	<0.001
BMI, kg/m^2^	19.91 ± 2.24	22.60 ± 2.35	24.50 ± 2.71	26.12 ± 3.08	<0.001
Current smoking, *N* (%)	539 (43.82%)	331 (26.85%)	256 (20.80%)	262 (21.27%)	<0.001
Current drinking, *N* (%)	393 (31.95%)	294 (23.84%)	263 (21.36%)	266 (21.59%)	<0.001
SBP, mmHg	147.01 ± 19.08	147.22 ± 17.56	146.97 ± 16.25	147.54 ± 17.18	0.850
DBP, mmHg	86.43 ± 11.22	88.86 ± 10.99	89.17 ± 10.59	91.15 ± 10.43	<0.001
Pulse rate, bpm	74.00 ± 15.98	75.44 ± 14.85	75.74 ± 13.52	78.49 ± 13.92	<0.001
Hcy, μmol/L	19.55 ± 11.63	19.05 ± 12.77	17.90 ± 11.00	17.41 ± 10.77	<0.001
FBG, mmol/L	5.69 ± 1.06	5.91 ± 1.13	6.17 ± 1.69	6.72 ± 2.11	<0.001
TC, mmol/L	4.79 ± 1.00	5.02 ± 1.02	5.28 ± 1.06	5.48 ± 1.19	<0.001
HDL-C, mmol/L	1.71 ± 0.44	1.53 ± 0.37	1.43 ± 0.33	1.31 ± 0.33	<0.001
LDL-C, mmol/L	2.50 ± 0.65	2.83 ± 0.70	3.13 ± 0.74	3.31 ± 0.81	<0.001
Uric acid, mmol/L	416.46 ± 115.47	416.62 ± 116.96	430.02 ± 120.91	463.09 ± 124.52	<0.001
eGFR, mL/min/1.73 m^2^	83.93 ± 19.91	85.24 ± 19.73	87.37 ± 18.68	88.55 ± 19.24	<0.001
Diabetes mellitus^$^	108 (8.78%)	175 (14.19%)	236 (19.17%)	376 (30.52%)	<0.001
Antihypertensive drugs	678 (55.12%)	743 (60.26%)	763 (61.98%)	787 (63.88%)	<0.001
Antiplatelet drugs	16 (1.30%)	35 (2.84%)	33 (2.68%)	24 (1.95%)	0.034
Glucose-lowering drugs	22 (1.79%)	40 (3.24%)	58 (4.71%)	81 (6.57%)	<0.001

*BMI, body mass index; SBP, systolic blood pressure; DBP, diastolic blood pressure; CHD, coronary heart disease; FPG, fasting plasma glucose; Hcy, homocysteine; HDL-C, high-density lipoprotein cholesterol; TC, total cholesterol; LDL-C, low-density lipoprotein cholesterol; eGFR, estimated glomerular filtration rate; FBG, fasting blood glucose*.

$ *diabetes mellitus was defined as self-reported physician diagnosis of diabetes or FBG concentration ≥7.0 mmol/l or use of glucose-lowering drugs*.

### Correlation Between the LAP Index and Cardiovascular Risk Factors

Table 2 shows the results of the Pearson's correlation analysis between the LAP index and cardiovascular risk factors. After age and sex adjustment, the LAP index was significantly correlated with BMI, SBP, DBP, FPG, TC, HDL-C, and LDL-C (*p* < 0.05).

**Table 2:** Correlation between the LAP index and cardiovascular risk factors after adjusted for age and sex.

	**LAP**	
	***r***	***P*-value**
BMI, kg/m^2^	0.663	<0.001
SBP, mmHg	0.035	0.011
DBP, mmHg	0.123	<0.001
FPG, mmol/L	0.232	<0.001
TC, mmol/L	0.186	<0.001
HDL-C, mmol/L	−0.400	<0.001
LDL-C, mmol/L	0.340	<0.001

*LAP, lipid accumulation product; BMI, body mass index; SBP, systolic blood pressure; DBP, diastolic blood pressure; FPG: fasting plasma glucose; HDL-C, high-density lipoprotein cholesterol; TC, total cholesterol; LDL-C, low-density lipoprotein*.

### Association of the LAP Index With baPWV and Elevated baPWV

Overall, the LAP index was found to be positively associated with baPWV in models 1–5. Values are the regression coefficients (β) of association, with model 1 adjusted only for age and sex; model 2 adjusted for prior covariates and BMI, SBP, DBP, pulse rate, pulse pressure, smoking status, and drinking status; model 3 adjusted for these prior covariates plus FBG, TC, HDL, LDL-C, Hcy, uric acid, eGFR, diabetes mellitus, antihypertensive drugs, and antiplatelet drugs; model 4 adjusted for these prior covariates plus the types of antihypertensive drugs and duration of hypertension; and model 5 adjusted for these prior covariates plus glucose-lowering drugs. Table 3 shows that in models 1–5, according to the β coefficients, for every 1-unit increase in ln LAP, the increases in baPWV were 19.81 (95% CI: 9.51, 30.11 cm/s), 42.39 (95% CI: 30.56, 54.22 cm/s), 25.06 (95% CI: 10.91, 39.22 cm/s), 24.12 (95% CI: 5.73, 42.51 cm/s), and 24.10 cm/s (95% CI: 5.72, 42.49 cm/s), respectively. As possible confounding factors were removed, the association became more significant. We also converted lnLAP from a continuous variable to a quartile variable. Compared with participants in the first quartile group, participants in quartile 2, quartile 3, and quartile 4 of the LAP index tended to have increased β coefficients of baPWV (*p* < 0.001) (Table 3). The results showed that LAP and baPWV had a positive linear correlation and were independent of cardiovascular risk factors.

**Table 3:** The association between LAP and brachial-ankle pulse wave velocity (baPWV) in different models.

**LAP index**	**baPWV, cm/s**, ***β*** **(95%CI)**				
	**Model 1**	**Model 2**	**Model 3**	**Model 4**	**Model 5**
Per 1 unit increase	19.81 (9.51, 30.11)	42.39 (30.56, 54.22)	25.06 (10.91, 39.22)	24.12 (5.73, 42.51)	24.10 (5.72, 42.49)
Quartiles					
Q1 (0.14 to <14.75)	0	0	0	0	0
Q2 (14.75 to <29.79)	46.38 (16.63, 76.14)	81.62 (54.37, 108.86)	70.29 (42.00, 98.59)	67.21 (29.84, 104.59)	67.34 (29.98, 104.70)
Q3 (29.79 to <52.83)	29.99 (-0.35, 60.33)	103.14 (72.52, 133.76)	84.00 (50.29, 117.72)	84.62 (40.03, 129.21)	84.51 (39.95, 129.08)
Q4 (52.83 to ≤ 514.60)	75.90 (44.75, 107.04)	150.06 (115.80, 184.32)	109.96 (69.73, 150.18)	115.95 (63.23, 168.68)	116.31 (63.61, 169.02)
*P* for trend	<0.001	<0.001	<0.001	<0.001	<0.001

*Model 1 adjusted only for age and sex*.

*Model 2 adjusted for prior covariates and BMI, SBP, DBP, pulse rate, pulse pressure, smoking status, and drinking status*.

*Model 3 adjusted for these prior covariates plus FBG, TC, HDL, LDL-C, Hcy, uric acid, eGFR, diabetes mellitus, antihypertensive drugs, and antiplatelet drugs*.

*Model 4 adjusted for these prior covariates plus the types of antihypertensive drugs and duration of hypertension*.

*Model 5 adjusted for these prior covariates plus glucose-lowering drugs*.

**Figure 1:**
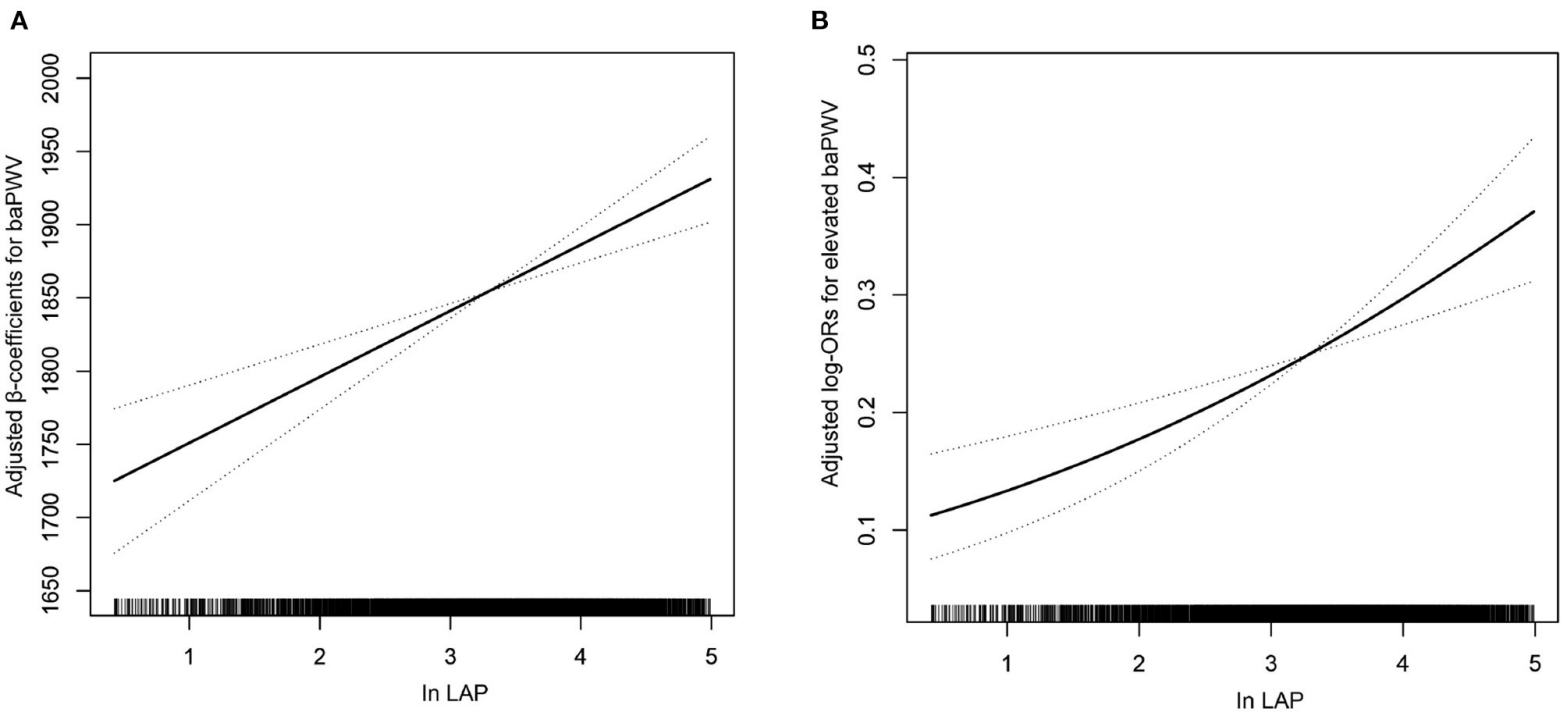
Association of the lipid accumulation product (LAP) index with **(A)** brachial-ankle pulse wave velocity (baPWV) and **(B)** elevated baPWV. A linear association between the LAP index and baPWV and elevated baPWV was found (*p* < 0.05). The solid line and dashed line represent the estimated values and their corresponding 95% CI. Adjustment factors included age, sex, body mass index (BMI), systolic blood pressure (SBP), diastolic blood pressure (DBP), pulse rate, pulse pressure, smoking status, drinking status, fasting blood glucose (FBG), total cholesterol (TC), high-density lipoprotein (HDL), low-density lipoprotein cholesterol (LDL-C), homocysteine (Hcy), uric acid, estimated glomerular filtration rate (eGFR), diabetes mellitus, antihypertensive drugs, antiplatelet drugs, the types of antihypertensive drugs, duration of hypertension, and glucose-lowering drugs.

As shown in Table 4, in a fully adjusted model 5, a positive association between the LAP and the risk of elevated baPWV was still existed. For every 1-unit increase in lnLAP, the adjusted ORs of elevated baPWV for participants in models 1–5 were 1.17 (95% CI: 1.09, 1.25), 1.42 (95% CI: 1.28, 1.58), 1.24 (95% CI: 1.09, 1.40), 1.19 (95% CI: 1.01, 1.41), and 1.19 (95% CI: 1.01, 1.41), respectively. When we converted lnLAP from a continuous variable to a quartile variable, the adjusted OR of elevated baPWV for participants in quartiles 2–4 was 1.41 (95% CI: 1.01, 1.96), 1.79 (95% CI: 1.20, 2.67), and 2.05 (95% CI: 1.28, 3.28) compared with participants in quartile 1 (*p* < 0.001). A generalized additive model and penalized spline method were used to further analyze the dose-response association between lnLAP and baPWV and elevated baPWV (Figures 1A,B), showing a significant positive linear association of the LAP index with baPWV and elevated baPWV. We conducted a sensitivity analysis to explore the association of the LAP index with baPWV and elevated baPWV in non-cancer participants and found that the results remained stable (Supplementary Tables 1, 2). Because there are gender differences in the calculation of the LAP index, we conducted a sensitivity analysis to explore the association of the LAP index with baPWV and elevated baPWV in different genders, the results are consistent with those in the general population (Supplementary Tables 5, 6).

**Table 4:** The association between LAP and elevated baPWV in different models.

**LAP index**	**Elevated baPWV, OR (95%CI)**				
	**Model 1**	**Model 2**	**Model 3**	**Model 4**	**Model 5**
Per 1 unit increase	1.17 (1.09, 1.25)	1.42 (1.28, 1.58)	1.24 (1.09, 1.40)	1.19 (1.01, 1.41)	1.19 (1.01, 1.41)
Quartiles					
Q1 (0.14 to <14.75)	1	1	1	1	1
Q2 (14.75 to <29.79)	1.27 (1.04, 1.55)	1.66 (1.31, 2.10)	1.51 (1.18, 1.93)	1.41 (1.01, 1.96)	1.41 (1.01, 1.96)
Q3 (29.79 to <52.83)	1.26 (1.02, 1.54)	2.16 (1.65, 2.83)	1.80 (1.33, 2.44)	1.79 (1.21, 2.67)	1.79 (1.20, 2.67)
Q4 (52.83 to ≤ 514.60)	1.71 (1.38, 2.11)	3.14 (2.32, 4.26)	2.24 (1.56, 3.21)	2.05 (1.28, 3.28)	2.05 (1.28, 3.28)
*P* for trend	<0.001	<0.001	<0.001	0.003	0.003

*Model 1 adjusted only for age and sex*.

*Model 2 adjusted for prior covariates and BMI, SBP, DBP, pulse rate, pulse pressure, smoking status, and drinking status*.

*Model 3 adjusted for these prior covariates plus FBG, TC, HDL, LDL-C, Hcy, uric acid, eGFR, diabetes mellitus, antihypertensive drugs, and antiplatelet drugs*.

*Model 4 adjusted for these prior covariates plus the types of antihypertensive drugs and duration of hypertension*.

*Model 5 adjusted for these prior covariates plus glucose-lowering drugs*.

### Subgroup Analyses by Potential Effect Modifiers

**Figure 2:**
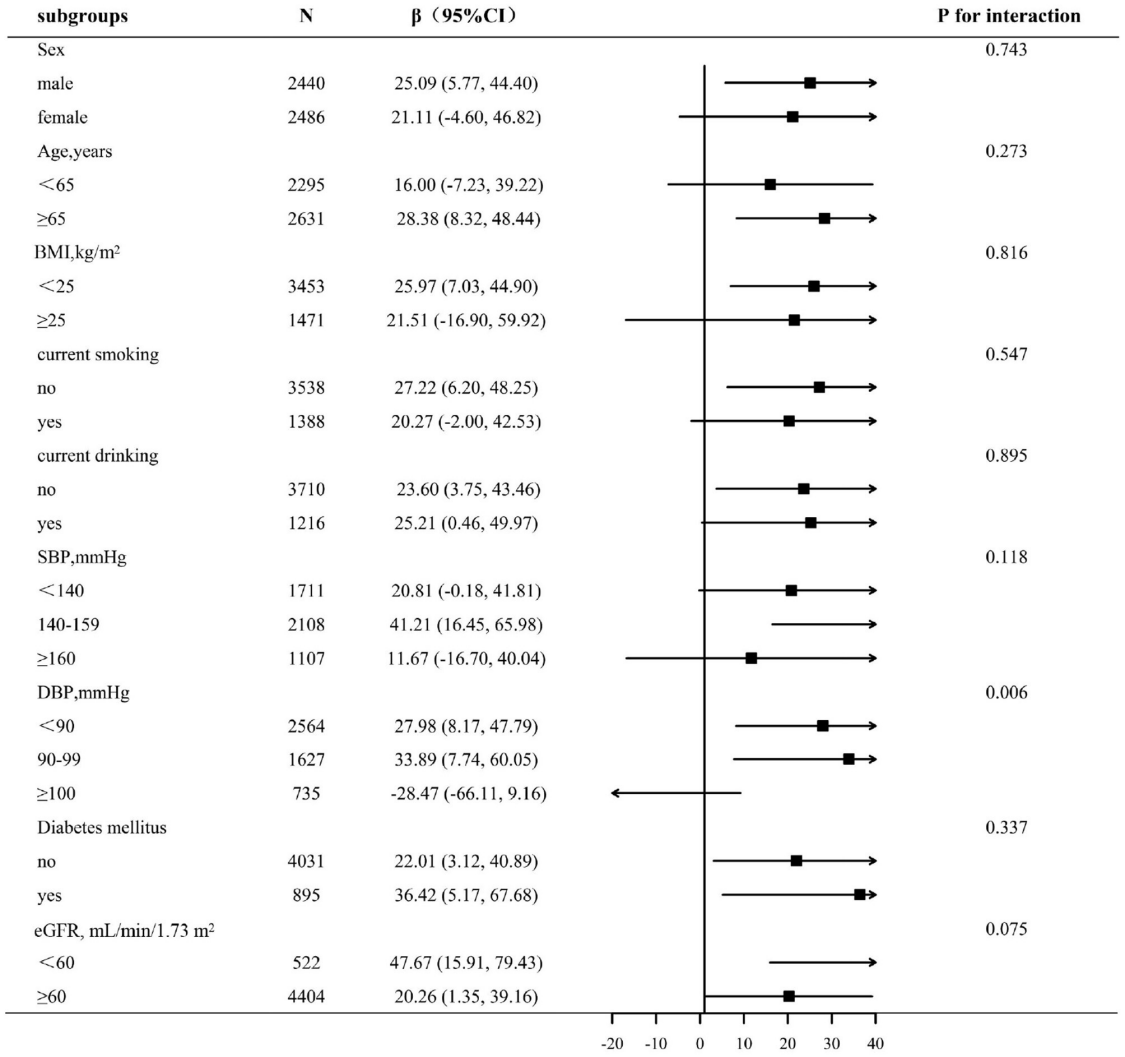
Subgroup analyses by potential effect modifiers. *Each subgroup analysis adjusted, if not stratified, for age, sex, BMI, SBP, DBP, pulse rate, pulse pressure, smoking status, drinking status, FBG, TC, HDL, LDL-C, Hcy, uric acid, eGFR, diabetes mellitus, antihypertensive drugs, antiplatelet drugs, the types of antihypertensive drugs, duration of hypertension, and glucose-lowering drugs.

To assess the relationship between lnLAP (per 1-unit increment) and baPWV in various groups, we performed exploratory subgroup analyses (Figure 2). There were no significant interactions in the following of the subgroup variables included sex (male vs. female; *p*-interaction = 0.808), age (<65 vs. ≥65 years; *p*-interaction = 0.587), BMI (<25 vs. ≥25 kg/m^2^; *p*-interaction = 0.568), current smoking (no vs. yes; *p*-interaction = 0.061), current drinking (no vs. yes; *p*-interaction = 0.861), SBP (<140, 140–159, ≥160 mm Hg; *p*-interaction = 0.283), diabetes mellitus (no vs. yes; *p*-interaction = 0.894), and eGFR (<60 vs. ≥60 ml/min/1.73 m^2^; *p*-interaction = 0.366). After adjustment for age, sex, BMI, SBP, DBP, pulse rate, pulse pressure, smoking status, drinking status, FBG, TC, HDL, LDL-C, Hcy, uric acid, eGFR, diabetes mellitus, antihypertensive drugs, antiplatelet drugs, the types of antihypertensive drugs, duration of hypertension, glucose-lowering drugs, except for the stratifying variables (*p* for interactions >0.05). Only in different subgroups of DBP, there is interaction between the LAP index and baPWV (DBP <90, 90–99, ≥100 mm Hg; *p*-interaction = 0.006). We found that the relationship between the LAP and baPWV was more significant among participants with DBP ≤ 99 mm Hg, DBP <90 mm Hg [27.98 (95% CI: 8.17, 47.79)], DBP = 90–99 mm Hg [33.89 (95% CI: 7.74, 60.05)], DBP ≥ 100 mm Hg [−28.47 (95% CI: −66.11, 9.16)].

## Discussion

This study includes 4,926 participants, which is the largest number of study on the same subject so far. In this relatively large-scale cross-sectional study, we found for the first time that the LAP index is independently positively correlated with baPWV and elevated baPWV in Chinese hypertensive patients and especially among participants with DBP ≤ 99 mm Hg.

The relation of the LAP index with baPWV has been evaluated in several previous studies and has yielded inconsistent results. The previous study has reported that the LAP index concerns the risk of arterial stiffness (18, 20) whereas some studies suggested that there was no association between the LAP index and arterial stiffness (19). Cicero et al. (18) conducted a study using the Brisighella Heart Study Database; a total of 1,731 healthy adults were included in this study; the mean age was 58.7 ± 15.8 years and 58.4 ± 15.5 years for men and women, respectively (*p* = 0.777). Moreover, the results showed that LAP was significantly correlated with the increase of baPWV [relative risk (RR) = 0.014, 95% CI: 0.008–0.020, *p* < 0.001]. A Japanese study explored the relationship between surrogate markers of insulin resistance including LAP and arteriosclerosis risk including 1,720 males (mean age: 38.8 ± 10.1 years) and 1,098 females (mean age: 39.1 ± 9.4 years) healthy adults. The results show that LAP is superior to other insulin resistance indexes including TyG and the LAP index is positively correlated with the elevated baPWV, which is more significant in women (20). Wakabayashi et al. (19) included 954 Japanese health examination subjects aged 39–64 years and found that the LAP index was not related to the increased risk of arterial stiffness. The reasons for the inconsistency of the above study results may be related to the characteristics of the study population, the size of the sample, and the definition of arterial stiffness. This study included 4,926 participants with an average age of 64.42 ± 9.44 years; the results indicated that the LAP index is positively correlated with baPWV and elevated baPWV and there is no interaction between the LAP index and baPWV in the gender subgroup. Sex and menopause-related changes in body composition and lipid metabolism (26, 27), which may lead to differences in the significance of surrogate markers of insulin resistance related to gender and menopause, may explain this finding. The women in this study population are basically in a post-menopausal state, so the prediction ability of the LAP index of women may decrease under estrogen level, which is similar to that of men. However, further study is needed to solve this hypothesis. The exact mechanism of the LAP index and arterial stiffness, especially in populations with hypertension, remains unclear. Studies have shown that LAP has a good correlation with insulin resistance and is considered as a surrogate marker of insulin resistance (IR) (28, 29). IR is closely related to the occurrence and development of atherosclerotic and is a risk factor of CVD (30). The specific pathological mechanism is as follows: it is generally believed that plasma insulin has a beneficial effect on vascular endothelial function (31) and vasodilation is induced by nitric oxide (NO) production accelerated by insulin receptor through phosphatidylinositol 3- kinase (PI3K)/Akt insulin signaling pathway (32). On the contrary, insulin resistance leads to the decrease of NO production by impairing PI3K/Akt signaling pathway (33), which is consistent with previous study results, which shows that insulin resistance is related to vascular dysfunction (34). The effect of insulin resistance on arterial stiffness includes an increase in sympathetic activity and the activation of the renin-angiotensin-aldosterone system. IR can put the body in a subclinical stress state, start the immune system slowly and slightly, and induce sustained and slight chronic inflammation, which leads to arterial stiffness (35, 36).

In order to better interpret the results of this study, we should include the limitations. First of all, this study is a cross-sectional study, so the causal relationship between LAP and baPWV cannot be obtained. Second, other confounding variables may affect our results, but regression analysis has adjusted many main parameters, so we are trustful in our observation. Finally, the population of this study is the relatively old hypertensive population in China, so it is limited to generalizability the results of this study to other populations.

## Conclusion

In summary, this large-scale cross-sectional study indicated a significant positive association between the LAP index and baPWV in Chinese adults with hypertension, especially among participants with DBP ≤ 99 mm Hg. In daily clinical practice, we should monitor the LAP index in hypertensive patients, especially those with DBP ≤ 99 mm Hg, which is helpful to prevent the occurrence of arterial stiffness and related CVD.

## Data Availability Statement

The datasets used and/or analyzed in the current study are available from the corresponding author upon reasonable request.

## Ethics Statement

The studies involving human participants were reviewed and approved by Ethics Committees of the Biomedical Institute of Anhui Medical University. The patients/participants provided their written informed consent to participate in this study.

## Author Contributions

YS participated in the literature search, data analysis, data interpretation, and wrote the manuscript. LH extracted and collected data. LH, ML, WZ, TW, LZ, and HB conceived the study and participated in its design and coordination. PL and XC participated in the study design and provided critical revision. All the authors read and approved the final version of the manuscript.

## Funding

This study was supported by the establishment and application of big data platform for clinical and scientific research management of hypertension in Jiangxi province (20172BCB22027), the central government guided local special funds for scientific and technological development (S2019CSFC0016), the Jiangxi Science and Technology Innovation Platform Project (20165BCD41005), and the National Key R&D Program of China (2018YFC1312902).

## Conflict of Interest

The authors declare that the research was conducted in the absence of any commercial or financial relationships that could be construed as a potential conflict of interest.

## Publisher's Note

All claims expressed in this article are solely those of the authors and do not necessarily represent those of their affiliated organizations, or those of the publisher, the editors and the reviewers. Any product that may be evaluated in this article, or claim that may be made by its manufacturer, is not guaranteed or endorsed by the publisher.
