# Supplementary material for: Relationship Between the Lipid Accumulation Product Index and Arterial Stiffness in the Chinese Population With Hypertension: A Report From the China H-Type Hypertension Registry Study
Source: Front Cardiovasc Med. 2022 Jan 21;8:760361. doi: 10.3389/fcvm.2021.760361 (PMC8823664; doi:10.3389/fcvm.2021.760361)
Supplement: Supplementary file 1 [file Data_Sheet_1.docx]

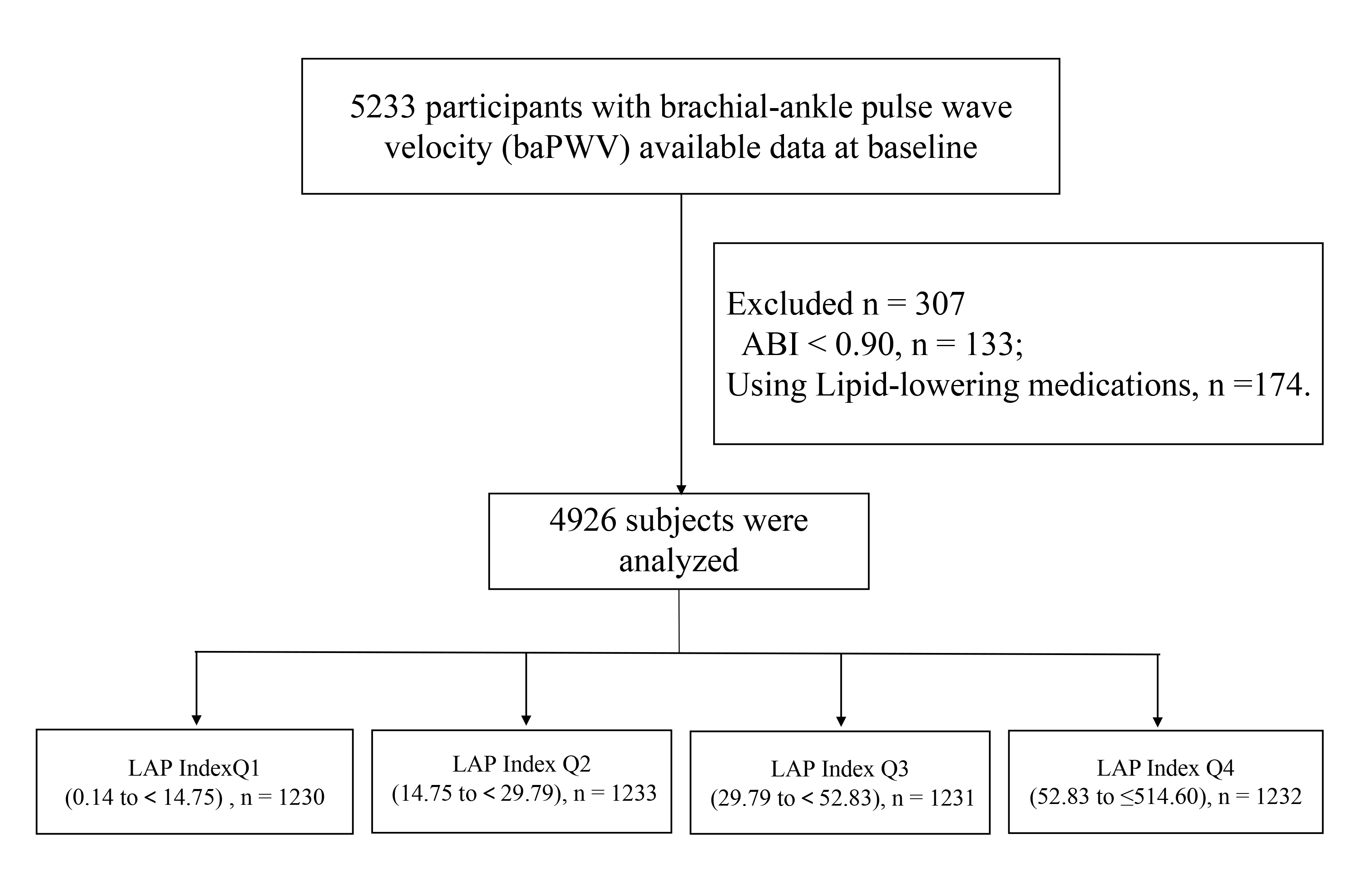


Figure S1 Flow chart of study participants

Supplementary Table 1 The association between LAP and baPWV in diferent models in non- cancer participants

| LAP Index | baPWV, cm/s, β (95%CI) | | |  |  |
| --- | --- | --- | --- | --- | --- |
|  | Model 1 | Model 2 | Model 3 | Model4 | Model5 |
| Per 1 unit increase | 19.63  (9.28, 29.99) | 42.54  (30.65, 54.43) | 24.63  (10.40, 38.86) | 24.00  (5.50, 42.50) | 24.01  (5.52, 42.51) |
| Quartiles |  |  |  |  |  |
| Q1 (0.14 to＜14.75) | 0 | 0 | 0 | 0 | 0 |
| Q2 (14.75 to＜29.79) | 43.39  (13.51, 73.27) | 79.62  (52.28, 106.96) | 67.85  (39.43, 96.28) | 66.58  (29.01, 104.15) | 66.71  (29.16, 104.26) |
| Q3 (29.79 to＜52.83) | 29.46  (-1.03, 59.95) | 102.98  (72.23, 133.74) | 82.62  (48.77, 116.47) | 84.24  (39.41, 129.06) | 84.21  (39.40, 129.02) |
| Q4 (52.83 to ≤514.60) | 75.37  (44.05, 106.68) | 150.40  (115.94, 184.86) | 109.02  (68.56, 149.49) | 116.23  (63.13, 169.33) | 116.66  (63.58, 169.74) |
| P for trend | <0.001 | <0.001 | <0.001 | <0.001 | <0.001 |

model1 adjusted only for age, sex;

model 2 adjusted for prior covariates and BMI, SBP, DBP, pulse rate, pulse pressure, smoking status, drinking status,

model 3 adjusted for these prior covariates plus FBG, TC, HDL, LDL-C, Hcy, uric acid, eGFR, diabetes mellitus, antihypertensive drugs, antiplatelet drugs,

model 4 adjusted for these prior covariates plus the types of antihypertensive drugs, duration of hypertension.

model 5 adjusted for these prior covariates plus glucose-lowering drugs.

Supplementary table 2 The association between LAP and elevated baPWV in diferent models in non- cancer participants

| LAP Index | Elevated baPWV, OR (95%CI) | | |  |  |
| --- | --- | --- | --- | --- | --- |
|  | Model 1 | Model 2 | Model 3 | Model4 | Model5 |
| Per 1 unit increase | 1.16 (1.08, 1.25) | 1.43 (1.28, 1.59) | 1.23 (1.09, 1.40) | 1.19 (1.01, 1.40) | 1.19 (1.01, 1.41) |
| Quartiles |  |  |  |  |  |
| Q1 (0.14 to＜14.75) | 1 | 1 | 1 | 1 | 1 |
| Q2 (14.75 to＜29.79) | 1.24 (1.02, 1.52) | 1.64 (1.29, 2.08) | 1.48 (1.15, 1.90) | 1.40 (1.00, 1.95) | 1.40 (1.00, 1.95) |
| Q3 (29.79 to＜52.83) | 1.24 (1.01, 1.52) | 2.15 (1.64, 2.82) | 1.78 (1.31, 2.40) | 1.81 (1.21, 2.70) | 1.81 (1.21, 2.70) |
| Q4 (52.83 to ≤514.60) | 1.69 (1.37, 2.10) | 3.17 (2.33, 4.30) | 2.22 (1.54, 3.19) | 2.04 (1.27, 3.28) | 2.04 (1.27, 3.28) |
| *P* for trend | <0.001 | <0.001 | <0.001 | 0.003 | 0.003 |

model1 adjusted only for age, sex;

model 2 adjusted for prior covariates and BMI, SBP, DBP, pulse rate, pulse pressure, smoking status, drinking status,

model 3 adjusted for these prior covariates plus FBG, TC, HDL, LDL-C, Hcy, uric acid, eGFR, diabetes mellitus, antihypertensive drugs, antiplatelet drugs,

model 4 adjusted for these prior covariates plus the types of antihypertensive drugs, duration of hypertension.

model 5 adjusted for these prior covariates plus glucose-lowering drugs.

Supplementary Table 3 Clinical characteristics of the study population according to LAP in males

| Variable | ln LAP | | | | *P* value |
| --- | --- | --- | --- | --- | --- |
|  | Quartile 1 | Quartile 2 | Quartile 3 | Quartile 4 |  |
| LAP range | 0.14 to＜14.76 | 14.76 to＜29.84 | 29.84 to＜52.93 | 52.93 to ≤514.60 | <0.001 |
| Participants | 865 | 620 | 501 | 454 |  |
| Age,year | 67.84 ± 8.56 | 64.75 ± 9.37 | 63.02 ± 9.80 | 58.93 ± 9.68 | <0.001 |
| BMI,kg/m^2^ | 20.11 ± 2.12 | 23.21 ± 2.22 | 25.04 ± 2.38 | 26.45 ± 2.80 | <0.001 |
| current smoking, N (%) | 498 (57.57%) | 281 (45.32%) | 205 (40.92%) | 222 (48.90%) | <0.001 |
| current drinking, N (%) | 362 (41.85%) | 247 (39.84%) | 206 (41.12%) | 220 (48.46%) | <0.001 |
| SBP, mmHg | 146.10 ± 19.50 | 145.70 ± 17.30 | 144.76 ± 16.25 | 145.00 ± 17.06 | 0.519 |
| DBP, mmHg | 86.97 ± 11.13 | 90.67 ± 11.10 | 91.33 ± 10.92 | 94.07 ± 10.35 | <0.001 |
| Pulse rate, bpm | 72.37 ± 14.67 | 74.02 ± 14.98 | 74.63 ± 14.66 | 77.52 ± 13.70 | <0.001 |
| Hcy,μmol/L | 21.05 ± 12.60 | 22.07 ± 15.60 | 20.23 ± 14.16 | 19.95 ± 13.75 | 0.053 |
| FPG, mmol/L | 5.64 ± 1.05 | 5.95 ± 1.16 | 6.22 ± 1.59 | 6.82 ± 2.38 | <0.001 |
| TC, mmol/L | 4.68 ± 0.95 | 4.86 ± 0.98 | 5.14 ± 1.07 | 5.31 ± 1.21 | <0.001 |
| HDL-C, mmol/L | 1.67 ± 0.44 | 1.46 ± 0.35 | 1.34 ± 0.31 | 1.22 ± 0.29 | <0.001 |
| LDL-C, mmol/L | 2.46 ± 0.63 | 2.76 ± 0.67 | 3.09 ± 0.73 | 3.24 ± 0.83 | <0.001 |
| Uric acid, mmol/L | 446.16 ± 109.94 | 463.69 ± 114.00 | 493.53 ± 119.82 | 532.21 ± 119.64 | <0.001 |
| eGFR, mL/min/1.73 m^2^ | 82.07 ± 20.79 | 83.75 ± 20.38 | 85.39 ± 19.67 | 87.25 ± 19.06 | <0.001 |
| Diabetes mellitus^$^ | 73 (8.44%) | 95 (15.32%) | 96 (19.16%) | 136 (29.96%) | <0.001 |
| Antihypertensive drugs | 498 (57.57%) | 397 (64.03%) | 321 (64.07%) | 289 (63.66%) | 0.022 |
| Antiplatelet drugs | 10 (1.16%) | 21 (3.39%) | 19 (3.79%) | 7 (1.54%) | 0.003 |
| Glucose-lowering drugs | 15 (1.73%) | 24 (3.87%) | 23 (4.59%) | 28 (6.17%) | <0.001 |

Abbreviation: BMI, body mass index; SBP, systolic blood pressure; DBP, diastolic blood pressure; CHD, coronary heart disease; FPG: fasting plasma glucose; Hcy, homocysteine; HDL-C, high-density lipoprotein cholesterol; TC, total cholesterol; LDL-C, low-density lipoprotein cholesterol; eGFR, estimated glomerular filtration rate.

^$^diabetes mellitus was defined as self-reported physician diagnosis of diabetes or FBG concentration ≥7.0 mmol/L or use of glucose-lowering drugs.

Supplementary Table 4 Clinical characteristics of the study population according to LAP in females

| Variable | ln LAP | | | | *P* value |
| --- | --- | --- | --- | --- | --- |
|  | Quartile 1 | Quartile 2 | Quartile 3 | Quartile 4 |  |
| LAP range | 0.34 to＜14.77 | 14.77 to＜29.81 | 29.81 to＜52.85 | 52.85 to ≤480.60 | <0.001 |
| Participants | 365 | 613 | 730 | 778 |  |
| Age,year | 66.60 ± 9.33 | 65.76 ± 9.67 | 64.42 ± 8.72 | 62.38 ± 8.44 | <0.001 |
| BMI,kg/m^2^ | 19.43 ± 2.42 | 21.97 ± 2.32 | 24.14 ± 2.86 | 25.93 ± 3.22 | <0.001 |
| current smoking, N (%) | 41 (11.23%) | 50 (8.16%) | 51 (6.99%) | 40 (5.14%) | 0.007 |
| current drinking, N (%) | 31 (8.49%) | 47 (7.67%) | 57 (7.81%) | 46 (5.91%) | 0.706 |
| SBP, mmHg | 149.18 ± 17.88 | 148.76 ± 17.70 | 148.49 ± 16.09 | 149.02 ± 17.09 | 0.907 |
| DBP, mmHg | 85.14 ± 11.34 | 87.03 ± 10.58 | 87.69 ± 10.09 | 89.44 ± 10.11 | <0.001 |
| Pulse rate, bpm | 77.86 ± 18.17 | 76.88 ± 14.60 | 76.50 ± 12.63 | 79.06 ± 14.02 | 0.003 |
| Hcy,μmol/L | 16.00 ± 7.87 | 16.00 ± 7.98 | 16.30 ± 7.77 | 15.92 ± 8.21 | 0.821 |
| FBG, mmol/L | 5.80 ± 1.07 | 5.87 ± 1.11 | 6.14 ± 1.75 | 6.67 ± 1.93 | <0.001 |
| TC, mmol/L | 5.05 ± 1.06 | 5.19 ± 1.03 | 5.38 ± 1.05 | 5.58 ± 1.17 | <0.001 |
| HDL-C, mmol/L | 1.79 ± 0.43 | 1.61 ± 0.37 | 1.49 ± 0.34 | 1.37 ± 0.34 | <0.001 |
| LDL-C, mmol/L | 2.62 ± 0.67 | 2.90 ± 0.72 | 3.15 ± 0.75 | 3.35 ± 0.80 | <0.001 |
| Uric acid, mmol/L | 346.06 ± 96.24 | 369.02 ± 99.40 | 386.44 ± 100.72 | 422.76 ± 108.62 | <0.001 |
| eGFR, mL/min/1.73 m^2^ | 88.33 ± 16.86 | 86.75 ± 18.95 | 88.73 ± 17.86 | 89.31 ± 19.31 | 0.072 |
| Diabetes mellitus^$^ | 35 (9.59%) | 80 (13.05%) | 140 (19.18%) | 240 (30.85%) | <0.001 |
| Antihypertensive drugs | 180 (49.32%) | 346 (56.44%) | 442 (60.55%) | 498 (64.01%) | <0.001 |
| Antiplatelet drugs | 6 (1.64%) | 14 (2.28%) | 14 (1.92%) | 17 (2.19%) | 0.896 |
| Glucose-lowering drugs | 7 (1.92%) | 16 (2.61%) | 35 (4.79%) | 53 (6.81%) | <0.001 |

Abbreviation: BMI, body mass index; SBP, systolic blood pressure; DBP, diastolic blood pressure; CHD, coronary heart disease; FPG: fasting plasma glucose; Hcy, homocysteine; HDL-C, high-density lipoprotein cholesterol; TC, total cholesterol; LDL-C, low-density lipoprotein cholesterol; eGFR, estimated glomerular filtration rate.

^$^diabetes mellitus was defined as self-reported physician diagnosis of diabetes or FBG concentration ≥7.0 mmol/L or use of glucose-lowering drugs.

Supplementary Table 5 The association between LAP and baPWV in diferent models in diferent sex

| LAP Index | baPWV, cm/s, *β* (95%CI) | | |  |
| --- | --- | --- | --- | --- |
|  | Model 1 | Model 2 | Model 3 | Model4 |
| male |  |  |  |  |
| Per 1 unit increase | 19.48  (6.62, 32.33) | 37.67  (21.79, 53.54) | 23.68  (5.12, 42.24) | 23.62  (5.09, 42.16) |
| Quartiles |  |  |  |  |
| Q1 (0.14 to＜14.75) | 0 | 0 | 0 | 0 |
| Q2 (14.75 to＜29.79) | 30.01  (-8.04, 68.05) | 64.92  (28.55, 101.28) | 56.04  (18.29, 93.80) | 55.45  (17.75, 93.15) |
| Q3 (29.79 to＜52.83) | 33.31  (-7.65, 74.28) | 103.33  (59.97, 146.68) | 91.26 (43.58, 138.94) | 90.80  (43.18, 138.41) |
| Q4 (52.83 to ≤514.60) | 84.12  (40.27, 127.98) | 149.25  (99.88, 198.62) | 115.44  (58.04, 172.84) | 115.93  (58.60, 173.25) |
| *P* for trend | <0.001 | <0.001 | <0.001 | <0.001 |
| female |  |  |  |  |
| Per 1 unit increase | 13.55  (-3.70, 30.80) | 44.64  (25.70, 63.58) | 24.37  (1.30, 47.43) | 24.53  (1.46, 47.60) |
| Quartiles |  |  |  |  |
| Q1 (0.34 to＜14.77) | 0 | 0 | 0 | 0 |
| Q2 (14.77 to＜29.81) | 50.89  (2.30, 99.48) | 94.27  (51.13, 137.41) | 84.29  (39.89, 128.69) | 84.47  (40.06, 128.87) |
| Q3 (29.81 to＜52.85) | 16.73  (-30.50, 63.97) | 98.46  (52.91, 144.00) | 77.55  (28.05, 127.04) | 77.52  (28.01, 127.02) |
| Q4 (52.85 to ≤480.60) | 60.45  (13.32, 107.58) | 143.64  (94.11, 193.18) | 102.37  (44.67, 160.07) | 102.53  (44.83, 160.23) |
| *P* for trend | 0.067 | <0.001 | 0.007 | 0.007 |

model1 adjusted only for age, sex;

model 2 adjusted for prior covariates and BMI, SBP, DBP, pulse rate, pulse pressure, smoking status, drinking status,

model 3 adjusted for these prior covariates plus FBG, TC, HDL, LDL-C, Hcy, uric acid, eGFR, diabetes mellitus, antihypertensive drugs, antiplatelet drugs,

model 4 adjusted for these prior covariates plus glucose-lowering drugs.

Supplementary Table 6 The association between LAP and elevated baPWV in diferent models in diferent sex

| LAP Index | Elevated baPWV, OR (95%CI) | | |  |
| --- | --- | --- | --- | --- |
|  | Model 1 | Model 2 | Model 3 | Model4 |
| male |  |  |  |  |
| Per 1 unit increase | 1.14 (1.05, 1.25) | 1.29 (1.12, 1.49) | 1.16 (0.98, 1.37) | 1.16 (0.98, 1.37) |
| Quartiles |  |  |  |  |
| Q1 (0.14 to＜14.75) | 1 | 1 | 1 | 1 |
| Q2 (14.75 to＜29.79) | 1.12 (0.87, 1.46) | 1.33 (0.96, 1.83) | 1.25 (0.88, 1.76) | 1.25 (0.88, 1.76) |
| Q3 (29.79 to＜52.83) | 1.22 (0.92, 1.62) | 1.78 (1.20, 2.63) | 1.63 (1.05, 2.55) | 1.63 (1.04, 2.55) |
| Q4 (52.83 to ≤514.60) | 1.70 (1.25, 2.31) | 2.58 (1.65, 4.03) | 1.92 (1.12, 3.27) | 1.92 (1.12, 3.28) |
| *P* for trend | 0.001 | <0.001 | 0.013 | 0.013 |
| female |  |  |  |  |
| Per 1 unit increase | 1.19 (1.06, 1.33) | 1.59 (1.35, 1.87) | 1.33 (1.09, 1.63) | 1.33 (1.09, 1.63) |
| Quartiles |  |  |  |  |
| Q1 (0.34 to＜14.77) | 1 | 1 | 1 | 1 |
| Q2 (14.77 to＜29.81) | 1.42 (1.04, 1.96) | 2.07 (1.43, 3.00) | 1.85 (1.26, 2.72) | 1.85 (1.26, 2.72) |
| Q3 (29.81 to＜52.85) | 1.32 (0.96, 1.80) | 2.60 (1.75, 3.86) | 2.03 (1.32, 3.13) | 2.03 (1.32, 3.13) |
| Q4 (52.85 to ≤480.60) | 1.78 (1.30, 2.44) | 3.81 (2.47, 5.87) | 2.55 (1.54, 4.22) | 2.54 (1.54, 4.22) |
| P for trend | 0.003 | <0.001 | 0.001 | 0.001 |

model1 adjusted only for age.

model 2 adjusted for prior covariates and BMI, SBP, DBP, pulse rate, pulse pressure,smoking status, drinking status,

model 3 adjusted for these prior covariates plus FBG, TC, HDL, LDL-C, Hcy, uric acid, eGFR, diabetes mellitus, antihypertensive drugs, antiplatelet drugs,

model 4 adjusted for these prior covariates plus glucose-lowering drugs.
